# H_2_O_2_ photoproduction inside H_2_O and H_2_O:O_2_ ices at 20–140 K

**DOI:** 10.1038/s41598-019-47915-w

**Published:** 2019-08-06

**Authors:** Mikhail Yu. Kulikov, Alexander M. Feigin, Otto Schrems

**Affiliations:** 1 Institute of Applied Physics of the Russian Academy of Sciences, 46 Ulyanov Str., 603950 Nizhny, Novgorod Russia; 2 Alfred Wegener Institute Helmholtz Centre for Polar and Marine Research, Am Handelshafen 12, D-27570 Bremerhaven, Germany

**Keywords:** Astrobiology, Photochemistry, Atmospheric chemistry, Geochemistry

## Abstract

We report the results of laboratory measurements of H_2_O_2_ production inside thin (50 nm thickness) H_2_O and H_2_O:O_2_ ice samples irradiated by 121.6 nm photons at different temperatures. In the case of H_2_O ice, H_2_O_2_ is formed at the temperatures below 60 К. In the case of H_2_O:O_2_ ice, H_2_O_2_ is formed in the 20–140 К range. For H_2_O:O_2_ = 9:1 ice, we derived H_2_O_2_ photochemical quantum yield as a function of sample irradiation temperature. The obtained data can be used for evaluation of H_2_O_2_ photoproduction at the surface of astrophysical water ice bodies and inside the particles of Noctilucent Clouds in the Earth’s atmosphere.

## Introduction

It is well known that surfaces of most icy bodies in the outer Solar System and interstellar space consist mainly of water and are regularly bombarded with energetic particles and photons. This irradiation triggers a spectrum of physicochemical processes inside solid phase^1–4^: the formation of primary products (H, OH, H_2_, and О); their fast recombination or leaving from the initial position with subsequent diffusion inside ice; reactions between them, with H_2_O or impurities; appearance of secondary products (HO_2_, HO_3_, H_2_O_2_, O_2_, O_3_); trapping of primary and secondary products by the ice matrix and their accumulation; and the flow of products into gas phase. The characteristics of these processes greatly depend on the characteristics of irradiation, ice type, its thickness, temperature, additives, and others. The end products such as H_2_O_2_, O_2_, O_3_, etc., both in solid and gas phase are of considerable interest for astrophysics as they are all oxidizing agents and may provide a source of chemical energy as a fuel for extraterrestrial life^5^.

The established detection of H_2_O_2_ on Europa’s surface^6^ and the discussed existence or absence of H_2_O_2_ on Enceladus, Ganymede, and Callisto^7^ stimulated extensive laboratory studies of the mechanisms of formation and measurement of the parameters of producing concentrations of this component in high-purity H_2_O ice and H_2_O ice with different additives irradiated by energetic particles^8–14^. In particular, it was shown that, as compared to H_2_O ice, the presence of О_2_ greatly increases the production of H_2_O_2_ and other products (HO_2_, HO_3_, and O_3_), especially at relatively high irradiation temperatures of 80–120 К. At the same time, except for a few works, significantly less attention was paid to investigation of H_2_O_2_ production by VUV photons. In particular, Gerakines *et al*.^15^ and Schriver *et al*.^16^ reported about H_2_O_2_ formation in H_2_O ice at 10 K irradiated by microwave discharge hydrogen flow lamps. Yabushita *et al*.^17^ found out H_2_O_2_ presence in H_2_O ice after its irradiation by 157 nm photons at 90 K. Shi *et al*.^18^ measured H_2_O_2_ in porous H_2_O + O_2_ ice irradiated by 193 nm photons at 40–78 K. Thus, many details of H_2_O_2_ production by VUV photons, the temperature dependence in the first place, are still understood poorly.

This work reports the first results of laboratory measurements of H_2_O_2_ production inside thin H_2_O and H_2_O:O_2_ ice samples irradiated by 121.6 nm photons in the temperature range of 20–140 K. We discuss possible implications of the obtained results at the surface of astrophysical water ice bodies and inside the particles of Noctilucent Clouds in the Earth’s atmosphere.

## Noctilucent Clouds

There exists at least one analog of such water icy bodies regularly irradiated by VUV photons in the Earth’s atmosphere. Each summer at polar and middle latitudes, one can observe the highest atmospheric clouds called Noctilucent Clouds (NLCs). They appear in mesopause region (altitudes range of 80–90 km) at the temperatures of 120–150 K^19–21^. Since the clouds discovery^22^, there were many discussions about their nature (see reviews by Gadsden & Schröder^19^ and by Thomas^20^). Only recently, the infrared spectra of clouds showed^23^ that NLCs consisted mainly of water ice. Thus, it is not doubt now that clouds form by condensation of water vapour and can influence on gas-phase chemistry of this region due to water vapour is its key parameter.

In the conditions of daytime mesopause, water vapour is subjected to intensive solar VUV radiation (121.6 nm, so called the Lyman–*α* line) and the reaction H_2_O + *hv* → H + OH provides the main chemical source of the family of odd hydrogen (HO_x_: H, OH, and HO_2_)^24^. In turn, the reactions with participation of HO_x_ components (O_3_ + H → O_2_ + OH, O + OH → O_2_ + H, O + HO_2_ → O_2_ + OH, and O_3_ + OH → O_2_ + HO_2_) remove the components of the odd oxygen family (O_x_: O(^1^D), O(^3^P), and O_3_). Therefore, the appearance of NLCs is expected to reduce the concentrations of water vapour and HO_x_ and to increase O_x_. However, rocket measurements of the concentration of atomic oxygen made during several campaigns demonstrated unpredicted O depletion around NLCs^25^. A possible explanation of the revealed effect was proposed by Murray and Plane^26^ who noticed that there also occurs photolysis of H_2_O molecules inside NLCs particles. The photoproducts (H and OH) may release into gas phase and additional source of HO_x_ leads to increase of O_x_ removal. In the work by Kulikov *et al*.^27^ this hypothesis was verified by laboratory measurements of the photodesorption rate from thin water ice samples irradiated by 121.6 nm photons in the temperature range of 120–150 K. It was found that most photoproducts did not leave the solid phase and tended to recombine in water molecules back. Basing on the results of ice irradiation by energetic particles at relatively high temperatures of 80–120 К^8–14^, we can assume that H_2_O_2_ is the principal photoproduct that may accumulate in NLCs.

Note that the experimental evidence of H_2_O_2_ concentration increase in the presence of clouds was obtained long ago. Arnold and Krankowsky^28^ presented results of several rocket mass-spectrometer measurements of H_2_O_2_^+^ ions above Andoya (Northern Norway, 69^○^ N) taken in different seasons. The model estimates of the concentration of those ions formed as a result of the H_2_O_2_ + O_2_^+^ → H_2_O_2_^+^ + O_2_ reaction agreed well with the results of measurements made in different seasons, except summer. The authors conjectured that the discrepancy was caused by the increased H_2_O_2_ concentration in the summer time. Later measurements^29^ revealed positively charged clusters containing H_2_O_2_ in the presence of NLCs particles. The researchers arrived at the conclusion that for such clusters to be formed, the concentration of H_2_O_2_ must be much higher than the expected level.

## Experimental Results

Hydrogen peroxide was found by detecting the IR absorption band of 2850–2860 cm^−1^ appeared in FTIR spectra of H_2_O and H_2_O:O_2_ ice samples as the result of their irradiation by calibrated source of 121.6 nm photons at high vacuum conditions. For more details please see Methods.

**Figure 1:**
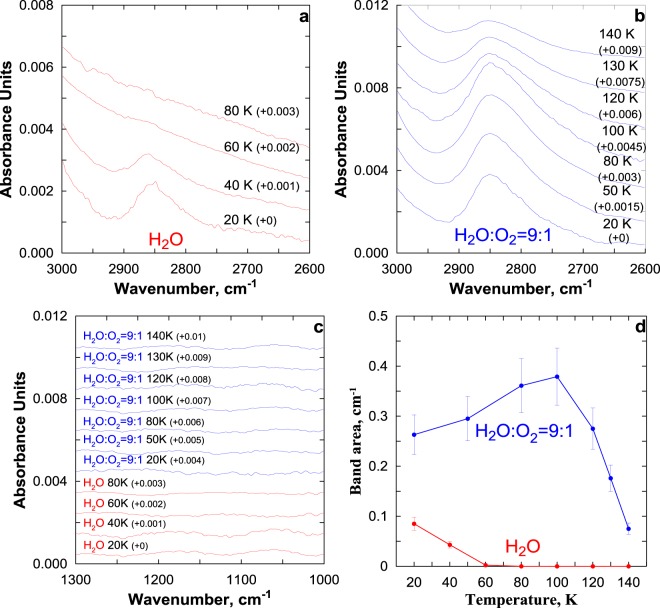
(**a**–**c**) Difference spectra (before and after irradiation) of H_2_O ice and H_2_O:O_2_ ice after 60 min of Lyman–*α* irradiation (*I*_*α*_ = 5 · 10^14^ photons/(cm^2^•s)) at different temperatures showing the temperature dependence of the appearance of H_2_O_2_ band near 2850–2860 cm^−1^ and the absent of new features at 1039, 1142, and 1259 cm^−1^ which can be attributed to O_3_, HO_2_, and HO_3_ correspondingly. Spectra above 20 K have been shifted vertically by an amount (shown in parentheses) to facilitate the display of the entire series. (**d**) Integrated area of the 2850–2860 cm^-1^ band as a function of temperature irradiation corresponding to (**a**,**b**).

The results of 1 hour irradiation of thin ice samples by Lyman-*α* photons at the photon flux intensity *I*_*α*_ = 5·10^14^ photons/(cm^2^·s) are presented in Fig. 1. One can see that, in the case of H_2_O ice (Fig. 1a), H_2_O_2_ is formed at temperature of irradiation (*T*_*ir*_) ≤ 60 К, and the temperature rise from 20 to 60 К results in a monotonic decrease of the integrated area of the 2850–2860 cm^−1^ band (${S}_{{H}_{2}{O}_{2}}$). At these temperatures, H_2_O_2_ peak position is at 2860 cm^−1^. The experiments with thin water ice samples doped with O_2_ give more interesting results (see Fig. 1b). First, H_2_O_2_ is formed in the entire studied temperature range of 20–140 К. Second, the temperature dependence of ${S}_{{H}_{2}{O}_{2}}$ is nonmonotonic, so that it attains its maximum (~0.38 ± 0.057) cm^−1^ at about 100 К. Third, typical values of ${S}_{{H}_{2}{O}_{2}}$ at 20–60 K are essentially higher that the maximum recorded in experiments with H_2_O ice at 20 К (~0.085 ± 0.013) cm^−1^. Forth, H_2_O_2_ peak position lays at 2850 cm^−1^ in the range of 20–100 K and shifts to 2852–2854 cm^−1^ at the temperatures 120–140 K. Note also, in both cases, there are no new features at 1039, 1142, and 1259 cm^−1^ (see Fig. 1c) which can be attributed to O_3_, HO_2_, and HO_3_ correspondingly^11,12^.

**Figure 2:**
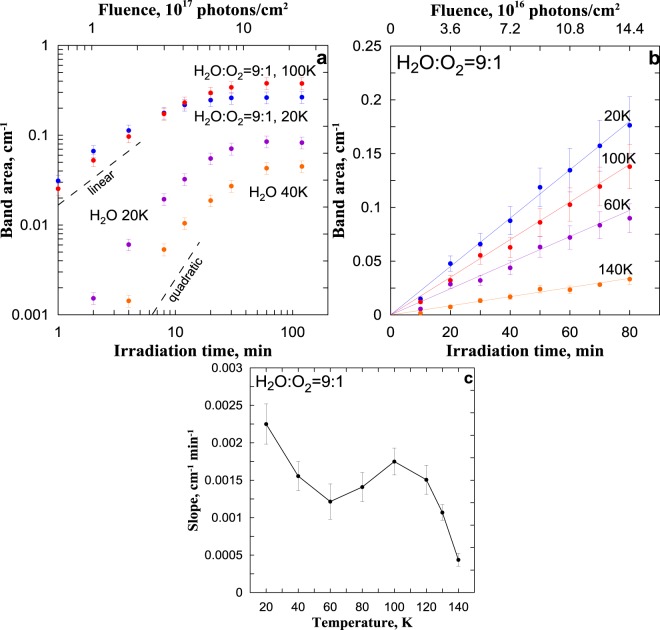
(**a**,**b**) Examples of integrated area of the 2850–2860 cm^-1^ band (color circles with bars) as a function of irradiation time (fluence) at *I*_*α*_ = 5·10^14^ photons/(cm^2^·s) (**a**) and at *I*_*α*_ = 3 · 10^13^ photons/(cm^2^·s) (**b**). Dashed lines in (**a**) indicate linear and quadratic fits. Color lines in (**b**) are linear fits. (**c**) Slope of the fit as a function of temperature irradiation corresponding to (**b**).

Figure 2a shows the examples of ${S}_{{H}_{2}{O}_{2}}$ dependencies on irradiation time (VUV fluence) at *I*_*α*_ = 5·10^14^ photons/(cm^2^·s). Note firstly, the present results are in a qualitative agreement with H_2_O_2_ temporal evolution obtained in earlier laboratory studies of the formation of this component in H_2_O ice irradiated by energetic particles^8,11,14^ and photons^15^. The temporal evolution of ${S}_{{H}_{2}{O}_{2}}$ can be divided into two parts: growth stage when ${S}_{{H}_{2}{O}_{2}}$ increases monotonically, and saturation stage. In both cases, ${S}_{{H}_{2}{O}_{2}}$ is saturated after ~1 hour irradiation. In the case of H_2_O ice, the growth stage continues for ~20–30 min and can be described by a quadratic function of irradiation time (VUV fluence). This corresponds to the results of irradiation of H_2_O ice by Lyman-*α* photons at 10 K obtained by Gerakines *et al*.^15^. In the case of H_2_O:O_2_ ice, the growth stage continues for ~10 min and can be described by a linear function of irradiation time (VUV fluence). Such behavior of ${S}_{{H}_{2}{O}_{2}}$ in the case of H_2_O:O_2_ ice was found at other photon flux intensities. It means that, in the case of H_2_O:O_2_ ice, the H_2_O_2_ production during the growth stage can be fit successfully to a (pseudo) first-order reaction. We can conclude that the rate of H_2_O_2_ production is proportional to *I*_*α*_ and can determine the H_2_O_2_ photochemical quantum yield (${\gamma }_{{H}_{2}{O}_{2}}$, the number of molecules of H_2_O_2_ generated per a Lyman-*α* photon absorbed by ice) as a function of *T*_*ir*_ following, for example, Cooper *et al*.^12^ and Hand and Carlson^14^. For this, we carried out special experiments with H_2_O:O_2_ ice at low*I*_*α*_ = 3·10^13^ photons/(cm^2^·s) at which the growth stage continued more than 100 min. The Fig. 2b shows the examples of ${S}_{{H}_{2}{O}_{2}}$ dependencies on irradiation time (fluence) at different *T*_*ir*_ with corresponding linear fits whose values of slope are presented on Fig. 2c.

### Estimation of H_2_O_2_ Column Density and H_2_O_2_ Photochemical Quantum Yield

**Figure 3:**
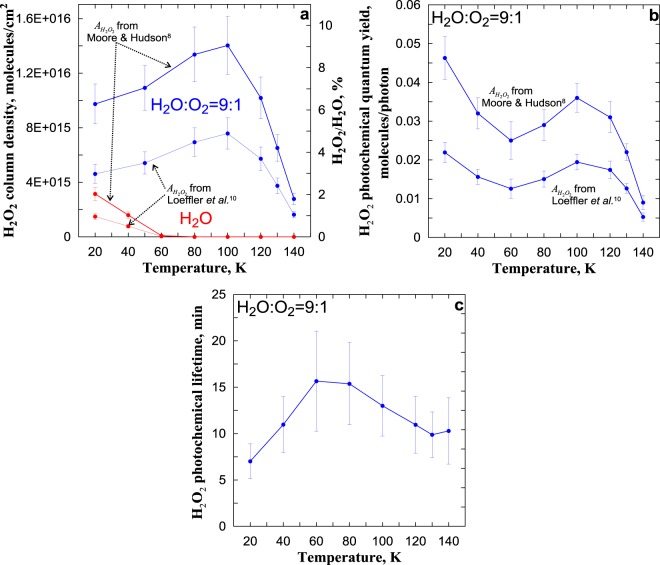
(**a**) H_2_O_2_ column density and its relative concentration in pure H_2_O ice and in H_2_O:O_2_ ice as a function of temperature irradiation corresponding to Fig. 1d. (**b**) H_2_O_2_ photochemical quantum yield as a function of temperature irradiation corresponding to Fig. 2c. (**c**) H_2_O_2_ photochemical lifetime as a function of temperature irradiation corresponding to (**a**,**b**).

All known studies of H_2_O_2_ production after irradiation of different ices^7,14,30–36^ used the values of ${A}_{{H}_{2}{O}_{2}}$ (the strength of the 2850–2860 cm^−1^ band absorption) from two sources^8,10^. Moore & Hudson^8^ measured ${A}_{{H}_{2}{O}_{2}}$ = 2.7·10^−17^ cm·molecule^−1^ and this value used for all temperatures. Loeffler *et al*.^10^ found out that ${A}_{{H}_{2}{O}_{2}}$ was 5.7·10^−17^ cm·molecule^−1^ at 20 K, 5.2·10^−17^ cm molecule^−1^ at 80 K, and 4.9·10^−17^ cm·molecule^−1^ at 110 K. Thus, for estimation of H_2_O_2_ column density (relative concentration) and H_2_O_2_ photochemical quantum yield from the results presented in Figs 1d and 2c, we applied both data sets (see Fig. 3a,b). In particular, Loeffler *et al*.^8^ data were interpolated into the temperature regions of 20–80 K and 80–110 K, and extrapolated to the temperature region of 110–140 K.

One can see from Fig. 3a, that, in the case of H_2_O ice, the maximum of relative concentration H_2_O_2_/H_2_O at 20 K is ~2% at ${A}_{{H}_{2}{O}_{2}}$ from Moore & Hudson^8^ and ~1% at ${A}_{{H}_{2}{O}_{2}}$ from Loeffler *et al*.^10^. In the case of H_2_O:O_2_ ice, the maximum of H_2_O_2_/H_2_O at 100 K is ~9% at ${A}_{{H}_{2}{O}_{2}}$ from Moore & Hudson^8^ and ~4.5% at ${A}_{{H}_{2}{O}_{2}}$ from Loeffler *et al*.^10^. In that time, the maximum of ${\gamma }_{{H}_{2}{O}_{2}}$ is at 20 K (see Fig. 3b). Note also, that at the temperatures of possible NLCs existence (120–140 K), ${\gamma }_{{H}_{2}{O}_{2}}$ varies within ~(0.009–0.031) molecules/photon at ${A}_{{H}_{2}{O}_{2}}$ from Moore & Hudson^8^ and ~(0.0053–0.0175) molecules/photon at ${A}_{{H}_{2}{O}_{2}}$ from Loeffler *et al*.^10^.

## Discussion and Conclusion

The possible mechanism of H_2_O_2_ formation during VUV irradiation of H_2_O ice was discussed by Gerakines *et al*.^15^ and pointed by Loeffler *et al*.^10^ in comparison with H_2_O_2_ formation by ions. This component is formed due to reaction OH + OH → H_2_O_2_. The quadratic character of ${S}_{{H}_{2}{O}_{2}}$ dependence on VUV fluence at the growth stage (see Fig. 2a) indicates a reaction of second order. It means that two photons are needed for appearance of one H_2_O_2_ molecule. These photons should produce two OH close to each other due to extremely low mobility of OH in water ice below 80 K^37,38^. The saturation of ${S}_{{H}_{2}{O}_{2}}$ after ~1 hour irradiation corresponds to the photochemical equilibrium when photoproduction is balanced by the photochemical sink due to the reactions H_2_O_2_ + *hv* → 2OH and H_2_O_2_ + OH → H_2_O + HO_2_.

In the case of H_2_O:O_2_ ice, the linear character of ${S}_{{H}_{2}{O}_{2}}$ dependence on VUV fluence at growth stage (see Fig. 2a) shows us that the mechanism of H_2_O_2_ formation differs from the previous case. Note, Loeffler *et al*.^10^ and Hand and Carlson^14^ found out the same behavior of ${S}_{{H}_{2}{O}_{2}}$ in H_2_O ice irradiated by high-energy ions and electrons correspondingly. It was proposed for explanation of this, in particular, that two OH could be produced in an ion track caused by an ion^10^. Evidently, this mechanism cannot be transferred on our situation. But, following Loeffler *et al*.^10^ and Hand and Carlson^14^, we can speculate that the rate of H_2_O_2_ formation in H_2_O:O_2_ ice irradiated by VUV photons is proportional to VUV intensity and the concentrations of H_2_O and O_2_. In other words, one photon, one H_2_O molecule and one O_2_ molecule are needed for appearance of one H_2_O_2_ molecule. On the other hand, O_2_ is impurity of H_2_O ice and typically two O_2_ molecules are separated from each other by a “paling” of H_2_O molecules. So, it is hard to imagine that these O_2_ molecules can participate in formation of one H_2_O_2_ molecule due to extremely low mobility of intermediates (such as OH and HO_2_) in water ice below 80 K as above mentioned. Thus, we can conclude as a first approximation that H_2_O_2_ photochemical quantum yield inside VUV irradiated H_2_O:O_2_ ice is proportional to the relative concentration of O_2_ (O_2_/H_2_O), when O_2_/H_2_O $\ll$ 1.

Note, that, in the case of H_2_O:O_2_ ice, temperature dependences of H_2_O_2_ column density (${N}_{{H}_{2}{O}_{2}}$) and ${\gamma }_{{H}_{2}{O}_{2}}$ shown in Fig. 3a,b allow to estimate the H_2_O_2_ photochemical lifetime (${\tau }_{{H}_{2}{O}_{2}}$) with the use of the photochemical equilibrium condition ${\tau }_{{H}_{2}{O}_{2}}\cdot {N}_{{H}_{2}{O}_{2}}={\gamma }_{{H}_{2}{O}_{2}}\cdot {I}_{\alpha }$. On can see from Fig. 3c that ${\tau }_{{H}_{2}{O}_{2}}$ has a maximum at 60–80 K and the temperature rise from 20 to 60 К results in an essential increase of ${\tau }_{{H}_{2}{O}_{2}}$. For explanation of this, we should take into account that H_2_O_2_ photochemical sink is due to the reactions (1) H_2_O_2_ + *hv* → 2OH and (2) H_2_O_2_ + OH → H_2_O + HO_2_. So, ${\tau }_{{H}_{2}{O}_{2}}={({R}_{1}+{R}_{2}\cdot OH)}^{-1}$, where *R*_1,2_ are the corresponding reaction rate coefficients. Note, Loeffler *et al*.^39^ measured the loss of H_2_O_2_ in H_2_O:H_2_O_2_ at temperatures between 21 and 145 K initiated by UV photons (193 nm). They obtained that the temperature dependences of H_2_O_2_ photodestruction cross section (${\sigma }_{{H}_{2}{O}_{2}}$) had a minimum at ~70 K which value was in ~5 and ~3 times more than ${\sigma }_{{H}_{2}{O}_{2}}$ at 20 K and 145 K correspondingly. Thus, following Loeffler *et al*.^39^, we can speculate that nonmonotonic temperature dependency of ${\sigma }_{{H}_{2}{O}_{2}}$ shown in Fig. 3c is caused by strong and nontrivial temperature dependency of cross section of H_2_O_2_ photodestruction by irradiation of our lamp which defines the value of *R*_1_. More detailed analysis of obtained results is out of the scopes of this short paper.

Thus, at the relatively high temperatures >60 К, H_2_O_2_ is photoproduced in H_2_O:O_2_ ice only. The estimated values of ${\gamma }_{{H}_{2}{O}_{2}}$ inside such ice can be used for assessing the impact of Lyman-*α* photons on water ice and its contribution to H_2_O_2_ production in different applications.

In astrophysics, VUV irradiation competes with energetic particles bombardment^3,40^. Moore and Hudson^8^ and Cooper *et al*.^12^ measured H_2_O_2_ production inside H_2_O:O_2_ = 6:1 ice mixtures irradiated with 0.8 MeV protons. It was found that G-value (defined as the number of H_2_O_2_ molecules created per unit of absorbed energy) varied in the range of 0.2–0.4 molecules/100 eV at 50–100 K, that is close to G-value of Lyman-*α* photons at 50–100 K obtained in our paper. Thus, contribution of Lyman-α photons to H_2_O_2_ production is defined by the ratio between energy fluxes of photons (*EF*_*ph*_) and energetic particles (*EF*_*ep*_). In the case of *EF*_*ph*_~*EF*_*ep*_, photons produce approximately the same amount of H_2_O_2_ inside ice as particles. At that, as it was noted by Gerakines *et al*.^40^, the penetration depth for Lyman-α photons (~45 nm^41^) in water ice is essentially less, than for protons which depends on its energy (for example, 1–2 μm for 0.1 MeV protons^10^ and 22 μm for 1 MeV protons^42^). So, one would expect the high relative H_2_O_2_ concentration in the top few tens of nm caused by Lyman-α photons. It means that H_2_O_2_ production by VUV photons can be important at *EF*_*ph*_/*EF*_*ep*_ ≥ 10^−2^.

In the mesopause region of the Earth’s atmosphere, typically *EF*_*ph*_ $\gg$ *EF*_*ep*_. But, at this moment, there is no information about containing of O_2_ inside water ice of Noctilucent Clouds. It is well-known that NLCs are formed as a result of gas-kinetic collision of H_2_O molecules with the surface of mesospheric aerosols, including adsorption and desorption properties. It is, generally, a relatively slow process, with the characteristic time (2–20 hours) depending on temperature^43^. In the real conditions of a summer mesopause, H_2_O concentration in gas phase is more than 4 orders of magnitude less than the concentration of O_2_ in ground (triplet) state and less than daytime concentration of O_2_ in singlet state ((2–4)·10^9^ cm^−3^ at 80–85 km^44^). The molecules of O_2_ in ground and excited states, like those of H_2_O, continually bombard the surface of the forming particles of clouds, adsorb on their surface and can be trapped inside the ice matrix as the NCL particles are being covered by new ice layers. This suggests that a small part of O_2_ molecules from gas phase may be uptaken by the forming matrix of the cloud. It is certainly hard to believe that the relative concentration of O_2_ inside NCL particles (${O}_{2}^{NLC}$) can be equal to 10% at such high temperatures. Nevertheless, we can estimate minimum of ${O}_{2}^{NLC}$ at which photoproduced H_2_O_2_ concentration would be comparable with the gas-phase concentration of this component.

Let us assume that ${\gamma }_{{H}_{2}{O}_{2}}^{NLC}$ is unknown H_2_O_2_ photochemical quantum yield in NCLs particles. Then the rate of H_2_O_2_ production per 1 cm^3^ at a certain altitude is defined by ${P}_{{H}_{2}{O}_{2}}={\gamma }_{{H}_{2}{O}_{2}}^{NLC}\cdot {S}_{Mie}\cdot {I}_{\alpha }$, where *I*_*α*_ is the local flux intensity of Lyman-α photons and *S*_*Mie*_ is the Mie absorption cross-section of NLCs, *S*_*Mie*_ ≈ *S*_*NLC*_/4, where *S*_*NLC*_ is the NLC surface density. According to the data of the long-term (1998–2005) measurements with the ALOMAR RMR-lidar in Northern Norway (69^○^ N, 16^○^ E), at the altitudes of 81–86 km *S*_*NLC*_ varies within the (3–6)·10^−8^ cm^2^/cm^3^ range^45^. Taking into consideration that at these heights in the conditions of average solar activity *I*_*α*_~3 · 10^11^ photons/(cm^2^·s)^24^, we obtain ${P}_{{H}_{2}{O}_{2}}$ ~ (2.25–4.5)·10^3^·${\gamma }_{{H}_{2}{O}_{2}}^{NLC}$ molecules/(cm^3^·s). In the real conditions of the mesopause, the NLCs particles are also irradiated by UV solar photons, which leads to H_2_O_2_ photodissociation in solid phase with the efficiency close to this process in gas phase^39^. For the considered range of altitudes of 81–86 km, the photodissociation constant of H_2_O_2_ in gas phase ${R}_{{H}_{2}{O}_{2}}$ is ~1.5 10^−4^ s^−1^. Thus, the equilibrium concentration of H_2_O_2_ that may be accumulated in cloud particles irradiated by Lyman-α photons is ${P}_{{H}_{2}{O}_{2}}/{R}_{{H}_{2}{O}_{2}}\,$ = (1.5–3)·10^7^·${\gamma }_{{H}_{2}{O}_{2}}^{NLC}$ cm^−3^. Gumbel *et al*.^25^ reported the maximum of *S*_*NLC*_~10^−7^ cm^2^/cm^3^, which gives the estimate for the maximum values of ${P}_{{H}_{2}{O}_{2}}$ and equilibrium concentration of H_2_O_2_ to be 7.5 · 10^3^·${\gamma }_{{H}_{2}{O}_{2}}^{NLC}$ molecules/(cm^3^·s) and 5 · 10^7^·${\gamma }_{{H}_{2}{O}_{2}}^{NLC}$ cm^−3^, correspondingly. Under the conditions of NLCs existence, typical value of gas-phase concentration of H_2_O_2_ is ~2 · 10^5^ cm^−3^ at 81 km and decreases with increasing height down to the values less than 10^4^ cm^−3^ at 86 km^26^. Thus, the minimum of ${\gamma }_{{H}_{2}{O}_{2}}^{NLC}$, at which photoproduced H_2_O_2_ concentration is equal to 10^4^ cm^−3^, corresponds to 2 · 10^−4^ molecules/photon. Following our above conclusions, H_2_O_2_ photochemical quantum yield inside H_2_O:O_2_ ice is proportional to the relative concentration of O_2_. Taking into consideration the measured values of ${\gamma }_{{H}_{2}{O}_{2}}$ inside H_2_O:O_2_ = 9:1 ice at the temperatures of 120–140 K, we estimate that the minimum of ${\gamma }_{{H}_{2}{O}_{2}}^{NLC}$ = 2·10^−4^ molecules/photon corresponds to ${O}_{2}^{NLC}$ ~ (0.065–0.22)% (i.e. the absolute concentration of O_2_ in NLCs ~(1.4–4.9)·10^5^ cm^−3^) at ${A}_{{H}_{2}{O}_{2}}$ from Moore & Hudson^8^ and ~(0.11–0.38)% (the absolute concentration ~(2.5–8.3)·10^5^ cm^−3^) at ${A}_{{H}_{2}{O}_{2}}$ from Loeffler *et al*.^10^. Note, that the estimated values of the O_2_ absolute concentration in NLCs are several orders of magnitude less than typical gas-phase concentrations of this component in ground and singlet states at the altitudes of 81–86 km.

To conclude, we have demonstrated for the first time that, if NLCs particles contain ≥0.1% O_2_, the physicochemical processes occurring in them may remarkably affect the chemical composition of the mesopause region. On the one hand, it may be a possible explanation of the results of early rocket mass-spectrometer measurements^28,29^ indicating increased H_2_O_2_ concentration in the clouds. On the other hand, H_2_O_2_, product of its UV photodissociation (OH), H_2_O, O_2_ and other impurities (for example, CO_2_) can participate in subsequent reactions producing more complex chemical compounds inside NLCs as it takes place, for example, in the bulk of supercooled water particles^46^. We hope that this research stimulates further experimental and theoretical investigations of the chemical composition of cloud particles. Note also that the obtained results are interesting for astrophysical applications, for example, for assessing the contribution of VUV irradiation to H_2_O_2_ production in the outer Solar System and interstellar space depending on temperature.

## Methods

### Apparatus

The experimental set-up was the same as we used under laboratory measurements of the photodesorption rate from water ice. As described by Kulikov *et al*.^27^, the apparatus consisted of a Fourier Transform Infrared Spectrometer (Bruker IFS 66 v)), a closed-cycle He refrigerator (Leybold ROK 10–300), a gas preparation and inlet system (further briefly GPIS), and a high-vacuum chamber with a volume of about 1000 cm^3^ pumped continuously by a turbomolecular pump system (Leybold-Heraeus) securing a high vacuum in the chamber down to the 10^−8^ mbar range. Inside the chamber, at the cold end of the cryostat there was a vertically mounted aluminium mirror (2.5 × 4 cm in size) as a substrate whose temperature was precisely regulated by a temperature controller (Lake Shore, model 340). The mirror temperature could be selected in the 10–300 K range. The GPIS was equipped with baratrons and needle valves. The upper part of the high vacuum chamber had two ports, one of which was equipped with a MgF_2_ (5 mm thick) input window for a VUV lamp. The second port had a KBr window for the IR beam of the FTIR spectrometer. The input for the VUV lamp made an angle of incidence of ~45^0^ to the mirror surface and, according to the estimates of the manufacturer, MgF_2_ transmitted about 60% of the quantum flux at the wavelength of 121.6 nm. As a VUV source (Lyman–*α*) we used a resonance hydrogen lamp (Opthos Instruments) containing a mixture of 10% H_2_ and 90% Ar excited by a microwave generator (Opthos Instruments, model MPG-4M) with a frequency of 2450 MHz. The lamp intensity was determined by the power supplied by the microwave generator (about the lamp calibration see below). The FTIR spectrometer was placed on rails allowing precise positioning of the instrument with respect to the cryostat with the sample. This was important for achieving a good overlap of the areas of the light spots from both, infrared (from spectrometer light source) and vacuum ultraviolet irradiation (from VUV lamp) of the ice film sample on the substrate. The operation of the FTIR spectrometer was PC controlled by means of software (OPUS) that permitted scanning spectra over a wide range (from 6000 to 500 cm^−1^) and analyzing the obtained spectra. The spectra were recorded with a spectral resolution of 0.2–2 cm^−1^ in the RAIRS mode (reflection absorption infrared spectroscopy) where the IR beam passes through the sample twice.

### Experimental procedures

The experimental procedures were almost the same as we used under laboratory measurements of the photodesorption rate from water ice. As described by Kulikov *et al*.^27^, each experiment with a particular sample of ice was conducted in two stages. At the first stage, two background spectra with different resolution were recorded at a mirror temperature of 20 K and at a temperature of subsequent irradiation (*T*_*ir*_). At that time, H_2_O or H_2_O + O_2_ gas was got ready in GPIS. We used the oxygen (Air Liquide 5.5) with purity better than 99.9995 Vol% and triply distilled water with resistivity better than 10^7^ ohm cm, additionally degassed by freeze/thaw cycles in vacuum conditions. As described in our previous study^27^, then, an ice film was prepared by depositing H_2_O or H_2_O + O_2_ vapour onto the mirror at 20 K. The deposition speed and sample thickness were controlled by a needle valve and internal baratron of GPIS and by tracking the evolution of absorption bands in the FTIR spectra. More specifically, a sample was prepared by successive slow depositions of small vapour portion which were accompanied by the integrated area measurements of the water ice 3275 cm^−1^ band (${S}_{{H}_{2}O}$). At the end of each sample deposition, we tried to rich the value of ${S}_{{H}_{2}O}\,$ = 31 ± 1 cm^−1^ which was equivalent to H_2_O column density ${N}_{{H}_{2}O}\,$ = (1.55 ± 0.05)·10^17^ molecules/cm^2^ at the strength of band absorption ${A}_{{H}_{2}O}\,$ = 2·10^−16^ cm/molecule taken from Allamandola *et al*.^47^. Here we applied the widely used^14,30,47^ approach to estimate a column density of absorbing molecules: ${N}_{{H}_{2}O}={S}_{{H}_{2}O}/{A}_{{H}_{2}O}$. In the case of H_2_O ice, the indicated values of ${N}_{{H}_{2}O}$ responded to thickness sample of 50 ± 1.6 nm at ice density of 0.93 g/cm^3^. In the case of H_2_O:O_2_ ice, we might expect increasing thickness of samples not more than 10%. Thus, the thicknesses of H_2_O and H_2_O:O_2_ ice samples were comparable with the attenuation depth (~45 nm^41^) of 10.2 eV photons in water ice and close to typical radii of NLC particles^48^. After the sample preparation, the mirror temperature was set at *T*_*ir*_ (in the 20–140 K range) and several IR spectra of unirradiated ice were recorded. At *T*_*ir*_ = 120 K and above, the IR spectra showed crystalline features of all ice samples that was in accordance with composition of NLCs^49^. Note also, Bartels-Rausch *et al*.^50^ discussed recently the simulations of disorder on pure ice at different temperatures below melting point (*T*_*m*_) and showed that, at the temperatures 10 K below *T*_*m*_, disorder affected the first molecular layer of ice only. In current study, the samples consisted of more than 100 layers of water and the highest temperature in our experiments (140 K) was about 20 K below than characteristic *T*_*m*_ of water ice in our vacuum chamber. Thus, we can conclude that interface processes could not influence essentially on the studied processes inside ice samples.

As described in our previous study^27^, at the second stage, the vacuum ultraviolet lamp was switched on and the ice films were exposed to VUV radiation with intensity set by microwave generator. After each photolysis period, IR spectra of the irradiated ice films were recorded. For improving the signal-to-noise ratio we used the spectral resolution of 2 cm^−1^ and a large amount of scans (2000). Hydrogen peroxide was found by detecting the IR absorption band of 2850–2860 cm^−1^ in difference spectra (before and after irradiation). The band was exuded by subtracting the baseline from the spectrum in manner described, for example, in Hand & Carlson^14^. Values of H_2_O_2_ column densities were determined as ${N}_{{H}_{2}{O}_{2}}={S}_{{H}_{2}{O}_{2}}/{A}_{{H}_{2}{O}_{2}}$, where ${S}_{{H}_{2}{O}_{2}}$ was the integrated area of the 2850–2860 cm^−1^ band, ${A}_{{H}_{2}{O}_{2}}$ was the strength of the band absorption.

### Calibration of the hydrogen discharge lamp

The calibration was carried out in the same manner as it was described by Kulikov *et al*.^27^. Before an experiment with specific H_2_O or H_2_O + O_2_ ice sample, we performed series of measurements of the absolute magnitude of the flux of Lyman-*α* photons that reach the ice sample at different adjustments of the microwave generator power. The widely used “ozone method”^16,51^ was applied for the procedure. The intensity of the lamp was determined by measuring the O_2_ → O_3_ conversion rate in a VUV photolyzed sample of solid O_2_ at 16 K. The ozone formation as a function of photolysis time was monitored with the FTIR spectrometer via the O_3_ absorption band at about 1040 cm^−1^. More specifically, for finding the VUV intensity at the mirror for a specific generator output power (GOP) we made successive measurements of the integrated area of the 1040 cm^−1^ absorption band (${S}_{{O}_{3}}$) as a function of irradiation time (see Fig. 2 in Kulikov *et al*.^27^). After that, the lamp intensity at this GOP was determined as ${I}_{\alpha }=d{S}_{{O}_{3}}/dt\cdot {(Y\cdot {A}_{{O}_{3}})}^{-1}$, where the derivative $d{S}_{{O}_{3}}/dt$ was found by the linear part of the function ${S}_{{O}_{3}}(t)$, *Y* was the quantum yield for the formation of O_3_ from O_2_, and ${A}_{{O}_{3}}$ was the strength of the band absorption. The value of $Y\cdot {A}_{{O}_{3}}$ was adopted from Cottin *et al*.^52^ and was equal to 8.4 · 10^−18^ cm·photon^−1^. We obtained that, depending on GOP varied within the range of 4–120 W, the photon flux intensity varied within the range 5 · 10^12^–10^15^ photons/(cm^2^•s). The stability of lamp intensity at the fixed GOP was checked by means of photodiode SXUV300 (International Radiation Detectors).
